# Advanced Readiness to Quit Smoking Among People Living With HIV Enrolled in a Smoking Cessation Trial in Hanoi, Vietnam: Associations With Risk Perception, Self-Efficacy, and Social Norms

**DOI:** 10.1177/1179173X261466277

**Published:** 2026-07-02

**Authors:** Thanh Ha-Lan Hoang, Gloria Guevara Alvarez, Nam Nguyen, Louise Adermark, Qinyun Lin

**Affiliations:** 1 School of Public Health and Community Medicine, Institute of Medicine, Sahlgrenska Academy, 70712 University of Gothenburg, Gothenburg, Sweden; 2 School of Global Public Health, 5894 New York University, New York, NY, USA; 3 Institute of Social and Medical Studies, Hanoi, Vietnam; 4 Department of Pharmacology, Institute of Neuroscience and Physiology, Sahlgrenska Academy, 195564 University of Gothenburg, Gothenburg, Sweden

**Keywords:** risk perception, readiness to quit, self-efficacy, social norm, HIV, smoking

## Abstract

**Background:**

In Vietnam, smoking remains highly prevalent among people living with HIV (PLWH), posing significant health risks. As PLWH receiving HIV care represent a selected population that may differ from the general smoking population, evidence on the psychosocial factors shaping readiness to quit in this group remains limited. This study examines the associations between tobacco-related risk perception, self-efficacy, and social norms with advanced readiness to quit among PLWH who smoke and were enrolled in a smoking cessation trial in Vietnam.

**Methods:**

We conducted a secondary cross-sectional analysis of baseline data from the VQUIT randomized controlled trial, including 672 PLWH who currently smoked cigarettes some days or every day, recruited from 13 HIV outpatient clinics in Hanoi, Vietnam. Advanced readiness to quit was defined as currently trying to quit or planning to quit within 30 days; all other responses were classified as lower or no readiness. Multivariable logistic regression models with robust standard errors, clustered at the clinic level, were fitted to examine associations between risk perception, self-efficacy, four social norm constructs, and advanced readiness to quit.

**Results:**

Overall, 76.6% of participants reported advanced readiness to quit. Higher risk perception was consistently associated with increased odds of advanced readiness to quit (aORs ranged 1.10 to 1.13 across models). Injunctive norms (aOR=1.11, 95% CI: 1.03–1.20) and internalized norms (aOR=1.40, 95% CI: 1.02–1.94) were positively associated with advanced readiness to quit, while descriptive and subjective norms were not. Self-efficacy showed no significant association.

**Conclusion:**

Among PLWH enrolled in a smoking cessation trial in Vietnam, higher risk perception and stronger injunctive and internalized norms were associated with advanced readiness to quit. Cessation interventions integrated into HIV care may benefit from addressing both perceived smoking-related risks and norm-based motivations to quit.

## Introduction

Tobacco use is a major driver of preventable morbidity and mortality among people living with HIV (PLWH), who experience substantially greater smoking-related harms than the general population.^1,2^ In the era of antiretroviral therapy (ART), several cohort and modelling studies have shown that PLWH who smoke may lose more life-years from smoking than from HIV itself, largely due to elevated risks of cardiovascular disease, respiratory illness, non-AIDS cancers, and premature death.^1,3,4^ Despite these risks, smoking prevalence among PLWH remains high in many low- and middle-income countries (LMICs). In Vietnam, national data from the Global Adult Tobacco Survey (GATS) indicate that smoking prevalence in the general adult population is around 22–23% overall, with a marked gender gap (about 45% in men and 1–2% in women).^5^ A large survey at outpatient clinics (OPCs) in Vietnam found that 36.1% of PLWH were current smokers, with prevalence far higher among men (59.7%) than women (2.6%).^6^ In addition, PLWH are less likely to quit smoking than the general population of people who use tobacco.^7,8^ Sociodemographic disparities, high nicotine dependence, pro-smoking social norms reinforcing smoking, and limited access to cessation treatment contribute to persistent smoking, while HIV-specific factors, including heightened stigma, mental health comorbidities, and chronic stress related to HIV, may further hinder cessation efforts among people living with HIV.^9,10^

Behavioral theories provide a useful framework for understanding motivation to quit smoking, including intention to quit smoking, which is a key proximal determinant of cessation as it predicts both subsequent quit attempts and the likelihood of achieving abstinence.^11,12^ The Health Belief Model posits that health-protective behaviors are influenced by risk perception, including perceived susceptibility to and severity of smoking-related harms.^13^ The Theory of Planned Behavior (TPB) proposes that behavioral intention is shaped by attitudes, self-efficacy (perceived behavioral control), and subjective norms (perceived expectations from important others).^14^

Building on these models, the Focus Theory of Normative Conduct distinguishes two externally oriented social norms: descriptive norms, or perceptions of how common smoking is among one’s peers, and injunctive norms, or perceived approval or disapproval of smoking.^15^ In addition, research on moral norms and smoking stigma highlights internalized (moral) norms, reflecting personal feelings of obligation, guilt, or moral concern related to smoking, as an additional source of motivation.^16,17^ Together with subjective norms from the TPB, these four norm constructs represent distinct pathways through which social influences may shape quitting motivation. Across these frameworks, risk perception, self-efficacy, and multiple dimensions of social norms may operate differently in Vietnam, where smoking is highly prevalent among men and where social relationships play an important role in shaping health behaviors.^5,18,19^

Despite the theoretical relevance of these constructs, empirical evidence from LMICs remains limited, particularly within HIV-care settings. Most studies among PLWH have focused on prevalence, sociodemographic or clinical correlates of smoking, with little examination of how psychosocial factors jointly influence quit motivation.^10^ This gap is notable in Vietnam, where no prior research has assessed these constructs simultaneously in PLWH engaged in HIV care. To address this, this study investigates the associations between risk perception, self-efficacy, and four dimensions of social norms (descriptive, injunctive, subjective, and internalized norms) and advanced readiness to quit cigarette smoking among PLWH receiving HIV care at OPCs in Vietnam. By elucidating how these psychosocial factors operate in a culturally specific and clinically important setting, the findings may inform the development of theory-informed, culturally sensitive cessation interventions tailored to PLWH in Vietnam and similar LMIC contexts.

## Methods

### Study Population and Data Source

This study is a secondary cross-sectional analysis of baseline data from the VQUIT trial that evaluated smoking cessation interventions among PLWH in Hanoi, Vietnam.^20^

Baseline data were collected between 30 November 2020 and 27 September 2023, with a sample size of 672 participants. Participants were screened for tobacco use at the time of registration for a routine visit. They were recruited from 13 HIV OPCs in Hanoi, and data were collected through in-person interviews. Eligible participants were adults aged 18 or older who currently smoked cigarettes or both cigarettes and waterpipes some days or every day, received care at OPCs, resided in Hanoi, and owned a mobile phone. Exclusion criteria included any contraindications to nicotine replacement therapy (NRT), such as recent myocardial infarction (within two weeks), significant arrhythmias, pregnancy, or breastfeeding. We also excluded those unable to provide informed consent or already engaged in a tobacco cessation program.

### Sample Size

The sample size was determined by the design of the parent trial, which was powered to evaluate intervention effects on smoking cessation outcomes. Because the current study is a secondary cross-sectional analysis of baseline data, no separate sample size calculation was conducted specifically for the present research question. Therefore, the findings should be interpreted in relation to the available baseline sample rather than as results from a study powered a priori for these observational associations.^21^

### Measures

Variable definitions and measurement procedures have been described in our previous analysis of the VQUIT baseline dataset and are briefly summarized here.^22^

#### Dependent Variable

Participants were asked to indicate their current intention to quit smoking using four response categories: **1** not planning to quit, **2** planning to quit within six months, **3** planning to quit within 30 days, and **4** currently trying to quit.

*Advanced readiness to quit* was operationalized as reporting either currently trying to quit or planning to quit within the next 30 days. Participants who reported planning to quit within six months or not planning to quit were classified as *having lower or no readiness*.

**Figure 1.**
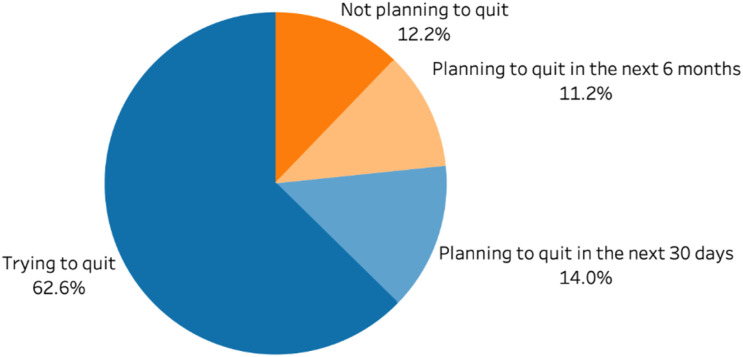
Distribution of original intention-to-quit categories prior to dichotomizing into advanced readiness versus low or no readiness to quit among study participants. Participants were classified into four categories: *Not planning to quit*, *planning to quit in the next 6 months*, *planning to quit in the next 30 days*, and *trying to quit*. Percentages represent the proportion of participants in each category. Data are based on 672 study participants

This classification was chosen to capture a near-action stage of quitting, reflecting immediate or imminent behavioral engagement rather than a full continuum of quit intention. Although planning to quit within six months may still reflect meaningful motivation, it represents a more distal stage of readiness compared with planning to quit within 30 days or actively trying to quit. In this trial-enrolled sample of PLWH who were already engaged in HIV care and a cessation program, most participants had at least some baseline motivation to quit. Therefore, focusing on more immediate readiness provided a clinically meaningful and analytically more stable distinction. Given the distribution of responses, dichotomizing the outcome also improved statistical balance and model stability, particularly by avoiding sparse categories that could lead to unstable estimates in models using the full ordinal scale. To address potential limitations of this categorization, we conducted sensitivity analyses using alternative outcome specifications. Figure 1 illustrates the original distribution of intention-to-quit categories.

#### Independent Variables

*Smoking-related risk perceptions* were assessed using four questions rated on a 4-point Likert scale, with response options ranging from 0-3 points corresponding to “Not at all”, “A little likely”, “Very likely” and “Extremely likely”, respectively: **1** “If you continue to smoke, how likely do you think it is that you will get a disease related to smoking like cancer, heart disease?”, **2** “How likely is it that continuing to smoke will increase your risk of getting an illness related to HIV?”, **3** ″In your opinion, how likely is it that quitting smoking would reduce your chances of getting a disease related to smoking like cancer or heart disease?”, **4.** “How worried are you about getting cancer, heart disease or other smoking-related diseases?”. A composite score was created by summing responses to all four items (range: 0–12), with higher scores indicating greater perceived risk.

*Self-efficacy* was measured using the Smoking Abstinence Self-efficacy Questionnaire (SASEQ) (range: 0-24).^23^

*Social norms* were conceptualized following established theoretical frameworks.

- **Descriptive norms** (perceptions of how common smoking is) were measured as the *number of smokers among the participant’s five closest friends* (range: 0–5). Higher scores indicate that smoking is more normative within the participant’s immediate peer group.
- **Injunctive norms** (perceived disapproval of smoking) were operationalized as the *number of people in the participant’s social network who object to their smoking* (range: 0–8). Higher values represent stronger perceived disapproval and social pressure to avoid smoking.
- **Subjective norms**, or perceived expectations from important others, were based on participants rating their agreement with the statement, *“People important to you believe that you should not smoke cigarettes”* (range: 1–4), using a 4-point Likert scale (1 = strongly disagree to 4 = strongly agree). Higher scores indicate stronger perceived expectations to quit smoking.
- **Internalized or moral norms**, including smoking-related stigma, were assessed by 4-point Likert scale questions (range: 1–4), where higher scores reflect greater self-perceived stigma or moral objection related to smoking. Participants were asked to indicate their level of agreement or disagreement with the following statements: (1) *I am embarrassed or ashamed that I am a smoker,* (2) *I am disappointed in myself for being a smoker,* and (3) *I feel inferior to others who are not smokers.*

#### Covariates

##### Sociodemographic Variables

Age in years, sex (*Male* or *Female*), education (*No school/Primary/Secondary school, High school (Grade 10-12)*, or *Vocational training/College/University*) and occupation (*Unemployed/Homemaker*, *Salaried/paid job*, or *Others*). Household income in the past 12 months in Vietnam Dong (VND) was categorized as high-income households (*≥300 million VND)* and lower-income households *(<300 million VND).*

##### Health-Related Variables

Depressive symptoms were assessed using Centre for Epidemiological Studies Depression Scale (CES-D 8, range: 0–24) and defined as *Yes* if CES-D 8 score ≥9 or *No*.^24,25^Self-rated health status was categorized as *Fair/Poor* or *Excellent/Very good/Good.*

##### Substance Use

Illicit drug use was classified as *Never, Not in the last 3 months* or *In the last 3 months* (if patients reported any illicit drug use such as cocaine, marijuana, heroin etc.). Hazardous/binge drinking was assessed using Alcohol Disorders Identification test (AUDIT-C) and adjusted for gender-specific thresholds (*Yes* if AUDIT-C score ≥ 4 for men and ≥3 for women or *No*).^26,27^

##### Tobacco Smoking-Related Variables

Nicotine dependence was based on Fagerström test of cigarette and waterpipe dependence (*Very low/Low/Medium* or *High/Very High*),^28,29^ and providers’ advice to quit at the last visit was captured (*No* or *Yes*).

### Statistical Analyses

The frequency distribution for each variable by advanced readiness to quit cigarettes was described. Baseline characteristics were compared descriptively between participants with advanced readiness versus lower or no readiness to quit. Multivariable logistic regression models were used to examine associations between advanced readiness to quit and risk perception, self-efficacy, and the different components of social norms, adjusting for covariates. Because participants were recruited from 13 OPCs, all regression models were estimated with robust standard errors clustered at the clinic level to account for potential intra-clinic correlation.

Since the four social norm constructs represent related but distinct theoretical dimensions of social influence, we estimated separate models for each construct to preserve interpretability and model parsimony. Pairwise correlations among the social norm constructs were examined, and an additional model including all four norm constructs simultaneously was estimated as a sensitivity analysis. Additionally, even after defining the outcome as advanced readiness to quit, the distribution remained highly imbalanced. Given the number of covariates included, combining all four social norm constructs would further reduce degrees of freedom and risk unstable estimates. We therefore estimated four separate models, each incorporating one norm construct together with risk perception, self-efficacy, and the same set of covariates. These covariates include age, education, employment, household income, healthcare provider advice to quit, nicotine dependence and drug use. Sex was not included as a covariate in the main models because the sample was overwhelmingly male (25 women (3.7%) out of 672 participants), resulting in insufficient variability to estimate stable sex-specific estimates within the logistic regression models. The odds ratios (ORs) with 95% confidence interval (95% CI) were estimated. To address missing values in low-income status, ten imputed datasets were generated using a logistic regression imputation model, and estimates were combined across imputations using Rubin’s rules.

We also performed several sensitivity analyses to assess the robustness of the findings to key modelling assumptions. First, we re-estimated the main regression models with additional adjustment for sex, binge drinking, self-rated health status, and depressive symptoms to evaluate the impact of extended covariate control. Second, since the outcome was relatively common in this sample, odds ratios may overestimate the magnitude of association. Therefore, we conducted a modified Poisson regression model with robust standard errors to estimate prevalence ratios (PRs) as an alternative effect measure. Finally, we estimated an additional model including all four social norm constructs simultaneously to assess whether the associations observed in the separate models were influenced by correlations among these theoretically related constructs.

All statistical tests were two-sided, and a p-value < 0.05 was considered statistically significant. We used STATA SE Version 18 to perform the statistical analyses.

## Results

### Descriptive Statistics

Table 1 presents the characteristics of the study participants. The study included 672 participants, of whom 77% had advanced readiness to quit. The median age was relatively similar across groups (43-44 years). Similarly, most participants in both groups were male (94-97%), had a high school education or higher (55-61%), were salaried employees (88-89%), and reported an annual household income below 300 million VND (85-88%). A large proportion in both groups also reported ever using illicit drugs (77-78%), hazardous alcohol intake (56-58%), and fair or poor self-rated health (68-71%).

**Table 1.** Study Participant Characteristics

Characteristics	Advanced readiness to quit			
	Advanced readiness n=515 (76.6%)	Lower or no readiness n=157 (23.4%)	Overall n=672	*p*-value
**SOCIODEMOGRAPHICS**				
Age (years)	44 [40,49]	43 [40,47]	44 [40,48]	0.127
Gender				
Male	499 (96.9%)	148 (94.3%)	647 (96.3%)	0.128
Female	16 (3.1%)	9 (5.7%)	25 (3.7%)	​
Education				
No school/Primary/Secondary school	233 (45.2%)	61 (38.9%)	294 (43.8%)	0.342
High school (Grade 10-12)	191 (37.1%)	63 (40.1%)	254 (37.8%)	​
Vocational training/College/University	91 (17.7%)	33 (21.0%)	124 (18.5%)	​
Occupation				
Unemployed/Homemaker	29 (5.6%)	9 (5.7%)	38 (5.7%)	0.975
Salaried/paid jobs	454 (88.2%)	139 (88.5%)	593 (88.2%)	​
Other (farmers, retired/students)	32 (6.2%)	9 (5.7%)	41 (6.1%)	​
Household income in past 12 months				
Equal to/More than 300 million VND	52 (10.1%)	21 (13.4%)	73 (10.9%)	0.252
Less than 300 million VND	455 (88.3%)	134 (85.3%)	589 (87.6%)	​
Missing	8 (1.6%)	2 (1.3%)	10 (1.5%)	​
**SUBSTANCE USE**				
Illicit drug use				
Never	118 (22.9%)	34 (21.7%)	152 (22.6%)	0.532
Not in the last 3 months	318 (61.7%)	93 (59.2%)	411 (61.2%)	​
In the last 3 months	79 (15.3%)	30 (19.1%)	109 (16.2%)	​
Hazardous drinking (AUDIT-C score ≥ 4 for men and ≥3 for women)				
No	217 (42.1%)	69 (43.9%)	286 (42.6%)	0.688
Yes	298 (57.9%)	88 (56.1%)	386 (57.4%)	​
**HEALTH**				
Depressive symptoms (CES-D 8)				
No	330 (64.1%)	92 (58.6%)	422 (62.8%)	0.214
Yes	185 (35.9%)	65 (41.4%)	250 (37.2%)	​
Self-rated health				
Excellent/Very good/Good	152 (29.5%)	51 (32.5%)	203 (30.2%)	0.478
Fair/Poor	363 (70.5%)	106 (67.5%)	469 (69.8%)	​
**TOBACCO SMOKING-RELATED VARIABLES**				
Risk Perception (Range: 0-12)	9 [7,11]	8 [6,10]	9 [7,10]	<0.001
Self-efficacy scores (SASEQ, Range: 0–24)	10 [7,14]	10 [8,13]	10 [7,14]	0.886
Social/moral norms				
Descriptive norms (Range: 0–5)	3 [2,5]	4 [2,5]	4 [2,5]	0.575
Injunctive norms (Range: 0–8)	3 [2,5]	2 [1,4]	3 [2,5]	<0.001
Subjective norms (Range:1–4)	3 [3,4]	3 [3,4]	3 [3,4]	0.004
Internalized norms (Range:1–4)	3 [2,3]	2 [2,3]	2 [2,3]	<0.001
Provider advised for tobacco use treatment at last visit				
Not advised	189 (36.7%)	71 (45.2%)	260 (38.7%)	0.055
Advised	326 (63.3%)	86 (54.8%)	412 (61.3%)	​
Fagerstrom Nicotine Dependence Category				
High/Very High	237 (46.0%)	67 (42.7%)	304 (45.2%)	0.461
Very low/Low/Medium	278 (54.0%)	90 (57.3%)	368 (54.8%)	​

*Note*. Median [IQR] was provided for continuous variables, and frequency (%) for categorical variables. Two-sample Wilcoxon rank-sum (Mann–Whitney) test used across levels of Advanced readiness to quit for continuous variables, and Pearson’s chi-squared test for categorical variables. *P*-values are presented for descriptive purposes only and were not used for formal hypothesis testing.

Depressive symptoms were common in both groups (36% among those with advanced readiness to quit and 41% among participants with lower or no readiness), as was high to very high nicotine dependence (46% vs. 43%, respectively). A higher proportion of participants with advanced readiness to quit reported receiving advice to quit smoking from a healthcare provider compared with those with lower or no readiness (63% vs. 55%).

Participants with advanced readiness to quit reported greater smoking-related risks than those with lower or no readiness. Stronger injunctive norms, indicating that more individuals in their social network disapproved of their smoking, as well as higher subjective and internalized (moral) norms related to smoking, were observed among participants with advanced readiness to quit. In contrast, descriptive norms, reflected by the number of close friends who smoke, and self-efficacy scores were similar across the two groups.

### Multivariable Logistic Regression Analysis

**Figure 2.**
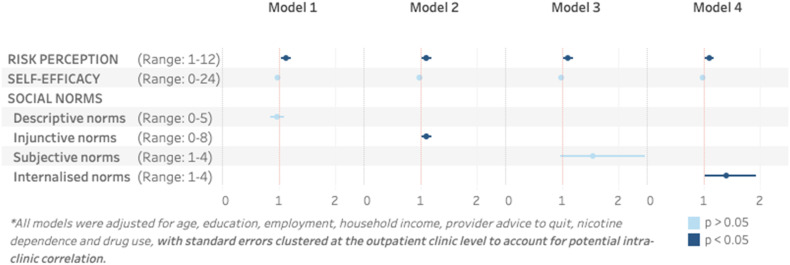
Associations of risk perception, self-efficacy, and social norms with advanced readiness to quit cigarette smoking (vs. lower readiness) across multivariable logistic models – primary analysis. Points represent adjusted effect estimates and horizontal lines indicate 95% confidence intervals. Vertical dashed lines indicate the null value. Separate models were fitted for each norm construct and adjusted for age, education, employment status, household income, provider advice to quit, nicotine dependence, and illicit drug use. Dark markers indicate statistically significant associations (p < 0.05), and light markers indicate non-significant associations (p ≥ 0.05). Scale ranges for each psychosocial measure are shown in parentheses. Standard errors were clustered at the outpatient clinic level to account for potential intra-clinic correlation

The results from the primary multivariable logistic regression analyses are presented in Table 2 and visualized in Figure 2. Across all four models incorporating one norm construct each, **higher risk perception** was consistently associated with greater odds of advanced readiness to quit (Model 1: aOR=1.13, 95% CI: 1.05–1.22, p=0.002; Model 2: aOR=1.11, 95% CI: 1.02–1.20, p=0.013; Model 3: aOR=1.11, 95% CI: 1.02–1.20, p=0.013; Model 4: aOR=1.10, 95% CI: 1.03–1.19, p=0.008). **Self-efficacy** was not significantly associated with advanced readiness in any model.

**Table 2.** Associations of Risk Perception, Self-Efficacy, and Social Norms With Advanced Readiness to Quit Cigarette Smoking (vs. Lower/No Readiness) Across Multivariable Logistic Models – Primary Analysis

​	Model 1		Model 2		Model 3		Model 4	
	OR (95% CI)	p-value	OR (95% CI)	p-value	OR (95% CI)	p-value	OR (95% CI)	p-value
RISK PERCEPTION	**1.13 (1.05–1.22)**	**0.002**	**1.11 (1.02–1.20)**	**0.013**	**1.11 (1.02–1.20)**	**0.013**	**1.10 (1.03–1.19)**	**0.008**
SELF-EFFICACY	0.98 (0.95–1.02)	0.392	0.99 (0.95–1.02)	0.470	0.99 (0.95–1.03)	0.554	0.99 (0.95–1.02)	0.447
SOCIAL NORMS								
Descriptive norms	0.97 (0.85–1.10)	0.616	​	​	​	​	​	​
Injunctive norms	​	​	**1.11 (1.03–1.20)**	**0.009**	​	​	​	​
Subjective norms	​	​	​	​	1.55 (0.97–2.47)	0.066	​	​
Internalized norms	​	​	​	​	​	​	**1.40 (1.02–1.94)**	**0.039**

*Bold cells highlight statistically significant associations.

Among the social norm variables, **descriptive norms**, reflecting the number of smoking friends, were not significantly associated with advanced readiness (Model 1: aOR=0.97, 95% CI: 0.85–1.10, p=0.616). In contrast, **injunctive norms** (aOR=1.11, 95% CI: 1.03–1.20, p=0.009) and **internalized norms** (aOR=1.40, 95% CI: 1.02–1.94, p=0.039) were positively associated with advanced readiness. Each additional person objecting to the participant’s smoking was associated with higher odds of advanced readiness. Stronger internalized norms were also associated with higher odds of advanced readiness. **Subjective norms** showed a positive but not statistically significant association with advanced readiness in the primary logistic model (aOR=1.55, 95% CI: 0.97–2.47, p=0.066).

We conducted several sensitivity analyses to assess the robustness of the findings. Overall, the findings were broadly consistent with the primary models. Results were similar after additional adjustment for sex, binge drinking, self-rated health, and depressive symptoms (Supplementary Table 1 and Supplementary Figure 1). Modified Poisson models produced smaller prevalence ratios than the corresponding odds ratios, as expected given the high prevalence of advanced readiness, but the direction of associations remained consistent (Supplementary Table 2 and Supplementary Figure 2). In the model including all four norm constructs simultaneously, norm-related estimates were attenuated after mutual adjustment (Supplementary Table 3 and Supplementary Figure 3). Given the weak-to-moderate correlations among these constructs (Supplementary Table 4), this attenuation likely reflects the estimation of independent associations among conceptually related dimensions of social norms. Additionally, analyses using the original ordinal outcome showed similar directional patterns but less precise estimates, with some violation of the proportional odds assumption and some convergence issues, supporting the use of the binary outcome specification and logistic regression as the primary analytic approach. These analyses supported risk perception as the most robust correlate of advanced readiness, with norm-related associations showing greater uncertainty.

## Discussion

This study examined factors associated with advanced readiness to quit smoking among PLWH enrolled in a smoking cessation trial and receiving care in OPCs in Vietnam. Across all models, higher risk perception was consistently associated with increased odds of advanced readiness to quit, while self-efficacy showed no significant association. Several dimensions of social norms, such as injunctive and internalized norms, were positively associated with advanced readiness to quit, while descriptive norms were not. Subjective norms also showed a positive association of similar magnitude, but the estimate was less precise and did not reach statistical significance in the primary logistic model. Together, these findings suggest that perceived smoking-related risk and selected normative processes, particularly perceived social disapproval and internalized moral concerns, may be relevant to advanced readiness to quit in this population. Given the cross-sectional design and selected trial-enrolled sample, the findings should be interpreted as associative and hypothesis-generating.

The positive association between risk perception and advanced readiness to quit aligns with the Health Belief Model, which suggests that perceived susceptibility and severity of health threats promote protective behaviors (e.g., quitting smoking).^13^ This association has been documented among studies on general population.^30,31^ Among PLWH specifically, studies have shown that awareness of heightened smoking-related risks is critical for cessation motivation.^32,33^ For instance, Pacek et al found that American PLWH who smoke perceive a greater risk of developing smoking-related disease, which correlated with intention to quit.^33^ Our findings extend this evidence into the Vietnamese HIV-care setting, indicating that strengthening risk awareness may be a key lever for enhancing advanced readiness to quit among PLWH who smoke and receive care in HIV OPCs.

In line with normative behavioral theories (such as the Theory of Planned Behavior and Focus Theory of Normative Conduct), injunctive norms and internalized norms were positively associated with advanced readiness to quit.^14,15^ These findings suggest that readiness to quit may be shaped not only by individual risk perception but also by perceived social disapproval of smoking and internalized moral concerns. Research among the general population suggests that perceived social expectations and moral evaluations influence quitting behavior.^34-36^ However, the salience of these normative influences may differ by context. In Vietnam’s collectivistic and male-smoking-normative culture, social approval, family expectations, and perceived responsibility toward others may be particularly relevant to smoking-related motivation. The positive association for internalized norms suggests that personal values, self-evaluation, shame, or disappointment related to smoking may also matter. However, such internalized responses should be interpreted carefully, as smoking-related stigma may motivate quitting for some individuals but may also contribute to shame, distress, or avoidance of care for others.^37,38^ Subjective norms showed a positive but non-significant association in the primary logistic model, although a similar association reached statistical significance in the modified Poisson sensitivity analysis. This uncertainty may partly reflect measurement limitations, as subjective norms were assessed using a single item.

In addition, the non-association of descriptive norms reflecting the number of smoking friends with advanced readiness to quit is notable. In our study, participants with advanced versus lower or no readiness to quit reported similar levels of descriptive norms (Table 1). This pattern suggests that when descriptive norms are nearly universal, their ability to discriminate readiness to quit may be structurally constrained. Some research finds that peer smoking influences smoking behavior in the general population^39,40^; however, in contexts where smoking is highly prevalent (such as among Vietnamese men), descriptive norms may have low variability and thus limited explanatory power. When almost everyone around a person smokes, perceiving that most friends smoke may not distinguish those ready to quit from those not ready. In contrast, other normative dimensions, such as injunctive norms (perceived disapproval to smoke from significant others) and internalized moral norms, may remain more salient and influential because they capture evaluative and motivational processes rather than mere prevalence, and thus better differentiate readiness to quit even in high-smoking environments.

Contrary to the Theory of Planned Behavior and much of the cessation literature, self-efficacy was not significantly associated with advanced readiness to quit in our sample. Although many studies among PLWH and the general population identify self-efficacy as a strong predictor of quit attempts and cessation success,^32,41,42^ some research has similarly reported no direct association with readiness to quit, instead suggesting that self-efficacy may operate differently across stages of the cessation process.^43,44^ Emerging evidence from HICs further indicates that the influence of self-efficacy may be dynamic and time-dependent rather than uniformly predictive at baseline.^45^ In population-based samples, baseline self-efficacy shows limited predictive value for cessation and readiness to quit, suggesting that it may not capture early motivational readiness.^41,46^Self-efficacy has been shown to fluctuate over short time scales and relate to daily abstinence intentions, supporting a more dynamic conceptualization than static baseline measurement alone.^47^

Methodological factors may also have contributed to the null finding in our study. Overall, the low self-efficacy scores in our sample (median ≈10 out of 24) and the restricted variability observed likely attenuated the association. Beyond measurement constraints, the sociocultural context offers further explanation. In Vietnam, smoking among men is highly prevalent and deeply embedded in social and cultural practices, including hospitality, male bonding, and workplace interactions.^5,18,19^ Within this context, readiness to quit may be shaped less by individual confidence alone and more by perceived risk, social disapproval, family responsibilities, and internalized concerns about smoking. Accordingly, injunctive and internalized moral norms reflecting responsibility toward one’s family and health, may exert a stronger motivational influence on readiness to quit than self-efficacy alone. Additionally, PLWH in Vietnam face layered structural and psychosocial challenges, including HIV-related stigma, depressive symptoms, economic hardship, and competing clinical demands that may reduce self-efficacy for quitting and shape how risks and norms are interpreted.^48,49^ Tobacco cessation interventions are not yet systematically integrated into routine HIV care, and few providers receive formal training in tobacco dependence treatment, despite OPCs representing a promising platform for cessation support.^20^ Taken together, these methodological and contextual factors likely explain why self-efficacy did not emerge as a significant correlate of advanced readiness to quit in this trial-enrolled sample.

## Strengths and Limitations

This study has several strengths. It used theory-informed measures of risk perception, self-efficacy, and multiple norm dimensions, including constructs rarely examined together in LMIC HIV-care settings. The study also drew on a relatively large sample of PLWH who smoke, recruited from multiple HIV OPCs in Hanoi. In addition, the analyses accounted for within-clinic correlation using robust standard errors clustered at the clinic level and included several sensitivity analyses, including modified Poisson models, alternative outcome specifications, additional covariate adjustment, and a model including all norm constructs simultaneously.

Nevertheless, several limitations should be considered. First, this was a secondary analysis of baseline data from participants enrolled in a smoking cessation trial. As most PLWH receiving HIV treatment in Vietnam are managed through OPCs, the OPC-based recruitment may reflect a major HIV-care setting in the country. However, participants in this study met the trial eligibility criteria and agreed to participate in a cessation intervention. They may therefore differ from PLWH who smoke but were ineligible, not reached, or unwilling to participate in cessation support. This should be considered when interpreting the generalizability of the findings. Second, self-reported measures may be affected by social desirability bias that leads to overreporting of readiness to quit smoking, particularly given their participation in a smoking cessation intervention trial. This context could have altered the observed association between risk perception, self-efficacy, social norms, and readiness to quit. Third, the cross-sectional design prevents us from establishing causality between these factors and advanced readiness to quit. Fourth, while our models adjusted for key confounders, residual confounding may still exist, particularly in unmeasured psychosocial factors influencing smoking behavior such as perceived barriers to quit and smoking cessation skills. In addition, although we used clinic-clustered standard errors to account for intra-clinic correlation, unmeasured contextual differences across OPCs, such as provider practices or cessation-support routines, may still have influenced the observed associations. Fifth, the dichotomization of the original intention-to-quit variable into advanced versus lower or no readiness may have reduced the granularity of the outcome and limited comparability with studies using multi-category intention measures. We explored models using the original ordinal outcome; however, these analyses showed less precise estimates and violation of the proportional odds assumption. Finally, the low representation of women also limited sex-stratified analyses; however, this distribution reflects the low prevalence of smoking among women living with HIV in Vietnam, suggesting that the sample is representative of the population most affected by tobacco use.

## Implications for Smoking Cessation Interventions & Future Research

These findings suggest several potential directions for tobacco cessation research and intervention development among PLWH in Vietnam. HIV OPCs may be important settings for cessation support because they provide regular contact with patients and are embedded in chronic HIV care. The robust association between risk perception and advanced readiness to quit suggests that provider-delivered communication about the HIV-specific and general health risks of smoking may be relevant for strengthening quit readiness.

The findings also suggest that social and moral dimensions of smoking may be important to consider. In particular, perceived disapproval from others and internalized concerns about smoking were associated with advanced readiness to quit. Future interventions could explore whether supportive, non-stigmatizing normative messages from healthcare providers, family members, or peers can strengthen advanced readiness to quit. However, approaches involving internalized norms should be designed carefully to avoid increasing shame or self-blame. Messaging should frame quitting as a positive and achievable health investment rather than as a moral failure.

Subjective norms showed a less precise association with advanced readiness, suggesting that perceived expectations from important others may still be relevant but require further study. Future work should use more detailed measures to distinguish expectations from family members, peers, and healthcare providers, and to examine how these sources of influence interact.

Given the lack of association between self-efficacy and advanced readiness in this baseline analysis, future studies should examine whether self-efficacy plays a stronger role at later stages of cessation, such as quit attempts, abstinence maintenance, or relapse prevention. Longitudinal research is needed to assess whether risk perception, self-efficacy, and social norm constructs predict actual quit attempts and sustained cessation over time.

Future studies should also include PLWH who smoke beyond cessation trial settings, particularly those who are ineligible, not reached, or unwilling to participate in cessation support, and participants from more diverse geographic settings in Vietnam. This would help clarify whether the psychosocial patterns observed in this trial-enrolled Hanoi sample apply to broader populations of PLWH who smoke.

## Conclusion

In this secondary baseline analysis of PLWH enrolled in a smoking cessation trial in Vietnam, higher smoking-related risk perception was consistently associated with advanced readiness to quit. Injunctive and internalized norms were also positively associated with advanced readiness. Self-efficacy and descriptive norms were not associated with advanced readiness, while subjective norms showed a positive but less precise association that did not reach conventional statistical significance. These findings suggest that perceived smoking-related risk, perceived social disapproval, and internalized concerns about smoking may be relevant correlates of near-term quit readiness among PLWH engaged in HIV care. Given the cross-sectional design and selected trial-enrolled sample, the findings should be interpreted as associative and hypothesis-generating. Future longitudinal and intervention studies are needed to examine whether these psychosocial factors predict quit attempts and cessation outcomes among broader populations of PLWH who smoke.

## Supplemental Material

Supplemental Material - Advanced Readiness to Quit Smoking Among People Living With HIV Enrolled in a Smoking Cessation Trial in Hanoi, Vietnam: Associations With Risk Perception, Self-Efficacy, and Social Norms Supplemental Material for Advanced Readiness to Quit Smoking Among People Living With HIV Enrolled in a Smoking Cessation Trial in Hanoi, Vietnam: Associations With Risk Perception, Self-Efficacy, and Social Norms by Thanh Ha-Lan Hoang, Gloria Guevara Alvarez, Nam Nguyen, Louise Adermark, and Qinyun Lin in Tobacco Use Insights.

## Data Availability

The datasets are available from the corresponding author upon reasonable request.*
