# Supplementary material for: Advanced Readiness to Quit Smoking Among People Living With HIV Enrolled in a Smoking Cessation Trial in Hanoi, Vietnam: Associations With Risk Perception, Self-Efficacy, and Social Norms
Source: Tob Use Insights. 2026 Jul 2;19:1179173X261466277. doi: 10.1177/1179173X261466277 (PMC13328995; doi:10.1177/1179173X261466277)
Supplement: Supplemental Material - Advanced Readiness to Quit Smoking Among People Living With HIV Enrolled in a Smoking Cessation Trial in Hanoi, Vietnam: Associations With Risk Perception, Self-Efficacy, and Social Norms [file sj-pdf-1-tui-10.1177_1179173X261466277.pdf]

Supplementary Figure/Table 1. **Associations of risk perception, self-efficacy, and social norms with advanced readiness to quit cigarette smoking (vs. lower readiness) across multivariable logistic models – Sensitivity analyses (Expanded covariate adjustment).** Points represent adjusted effect estimates (odds ratios) and horizontal lines indicate 95% confidence intervals. Vertical dashed lines indicate the null value. Separate models were fitted for each norm construct and adjusted for age, sex, education, employment status, household income, provider advice to quit, nicotine dependence, illicit drug use, binge drinking, self-rated health status and depressive symptoms. Dark markers indicate statistically significant associations ($p < 0.05$), and light markers indicate non-significant associations ($p \geq 0.05$). Scale ranges for each psychosocial measure are shown in parentheses.

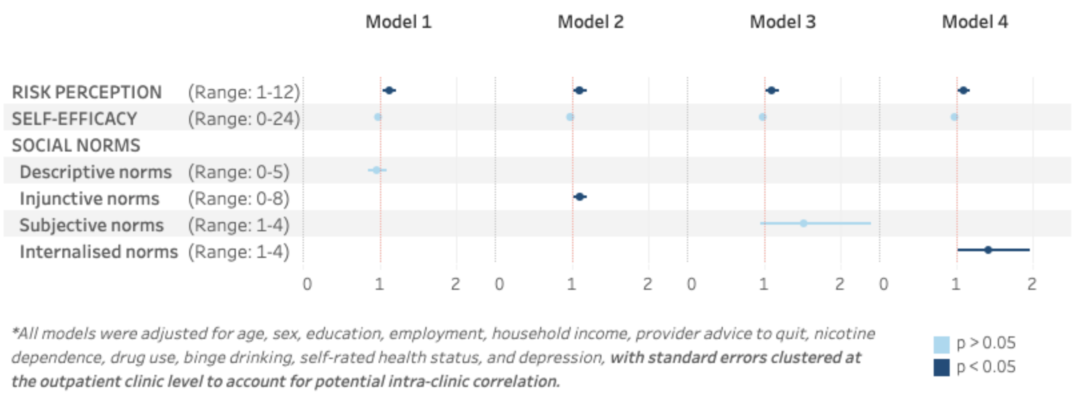

	Model 1		Model 2		Model 3		Model 4	
	**OR (95% CI)**	**p-value**	**OR (95% CI)**	**p-value**	**OR (95% CI)**	**p-value**	**OR (95% CI)**	**p-value**
**RISK PERCEPTION**	**1.13 (1.04–1.22)**	**0.002**	**1.10 (1.02–1.19)**	**0.017**	**1.10 (1.02–1.19)**	**0.017**	**1.10 (1.02–1.18)**	**0.010**
**SELF-EFFICACY**	0.98 (0.95–1.02)	0.296	0.98 (0.95–1.02)	0.360	0.99 (0.95–1.02)	0.466	0.98 (0.95–1.02)	0.336
**SOCIAL NORMS**								
**Descriptive norms**	0.96 (0.85–1.09)	0.561						
**Injunctive norms**			**1.11 (1.02–1.20)**	**0.013**				
**Subjective norms**					1.52 (0.96–2.40)	0.073		
**Internalised norms**							**1.42 (1.03–1.96)**	**0.033**

Supplementary Figure/Table 2. **Associations of risk perception, self-efficacy, and social norms with advanced readiness to quit cigarette smoking (vs. lower readiness) across multivariable models – Sensitivity analyses (Poisson models).** Points represent adjusted effect estimates (prevalence ratios) and horizontal lines indicate 95% confidence intervals. Vertical dashed lines indicate the null value. Separate models were fitted for each norm construct and adjusted for age, education, employment status, household income, provider advice to quit, nicotine dependence, and illicit drug use. Dark markers indicate statistically significant associations ($p < 0.05$), and light markers indicate non-significant associations ($p \geq 0.05$). Scale ranges for each psychosocial measure are shown in parentheses.

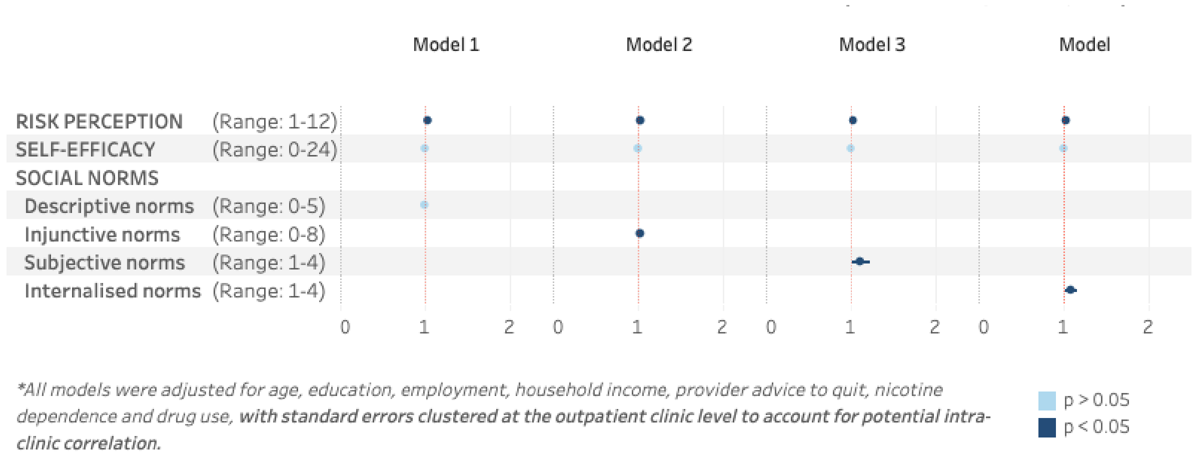

	**Model 1**		**Model 2**		**Model 3**		**Model 4**	
	**PR (95% CI)**	**p-value**	**PR (95% CI)**	**p-value**	**PR (95% CI)**	**p-value**	**PR (95% CI)**	**p-value**
**RISK PERCEPTION**	**1.03 (1.01–1.05)**	**0.002**	**1.02 (1.01–1.04)**	**0.011**	**1.02 (1.01–1.04)**	**0.010**	**1.02 (1.00–1.04)**	**0.018**
**SELF-EFFICACY**	1.00 (0.99–1.01)	0.399	1.00 (0.99–1.01)	0.402	1.00 (0.99–1.01)	0.452	1.00 (0.99–1.01)	0.407
**SOCIAL NORMS**								
**Descriptive norms**	0.99 (0.97–1.02)	0.599						
**Injunctive norms**			**1.02 (1.00–1.04)**	**0.017**				
**Subjective norms**					**1.11 (1.02–1.20)**	**0.015**		
**Internalised norms**							**1.08 (1.01–1.16)**	**0.036**

Supplementary Figure/Table 3. **Associations of risk perception, self-efficacy, and social norms with advanced readiness to quit cigarette smoking (vs. lower readiness) – Sensitivity analyses (All four social norm constructs in one model).** Points represent adjusted effect estimates (odds ratios) and horizontal lines indicate 95% confidence intervals. Vertical dashed lines indicate the null value. The multivariable logistic model was adjusted for age, education, household income, provider advice to quit, nicotine dependence, and illicit drug use. Dark markers indicate statistically significant associations ($p < 0.05$), and light markers indicate non-significant associations ($p \geq 0.05$). Scale ranges for each psychosocial measure are shown in parentheses. Standard errors were clustered at the outpatient clinic level to account for potential intra-clinic correlation.

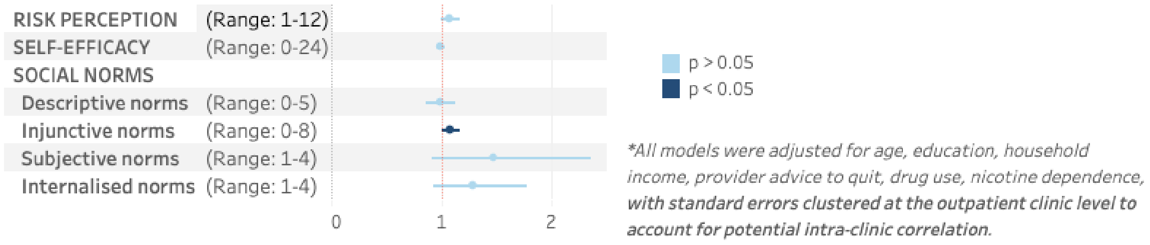

	Model 1	
	**OR (95% CI)**	**p-value**
**RISK PERCEPTION**	1.07 (0.99–1.16)	0.099
**SELF-EFFICACY**	0.99 (0.95–1.03)	0.509
**SOCIAL NORMS**		
**Descriptive norms**	0.99 (0.86–1.13)	0.851
**Injunctive norms**	**1.08 (1.00–1.16)**	**0.050**
**Subjective norms**	1.47 (0.92–2.35)	0.110
**Internalised norms**	1.28 (0.93–1.77)	0.130

Supplementary Table 4. Correlations among social norm constructs

	Descriptive norms	Injunctive norms	Subjective norms	Internalised norms
**Descriptive norms**	1.000			
**Injunctive norms**	-0.125	1.000		
**Subjective norms**	-0.022	0.168	1.000	
**Internalised norms**	-0.070	0.329	0.152	1.000
